# *In Vivo* Imaging and Quantitative Analysis of Leukocyte Directional Migration and Polarization in Inflamed Tissue

**DOI:** 10.1371/journal.pone.0004693

**Published:** 2009-03-04

**Authors:** Alexander Georg Khandoga, Andrej Khandoga, Christoph Andreas Reichel, Peter Bihari, Markus Rehberg, Fritz Krombach

**Affiliations:** 1 Walter Brendel Centre of Experimental Medicine, Ludwig-Maximilians-Universität München, Munich, Germany; 2 Department of Surgery-Grosshadern, Ludwig-Maximilians-Universität München, Munich, Germany; University of Nebraska, United States of America

## Abstract

Directional migration of transmigrated leukocytes to the site of injury is a central event in the inflammatory response. Here, we present an *in vivo* chemotaxis assay enabling the visualization and quantitative analysis of subtype-specific directional motility and polarization of leukocytes in their natural 3D microenvironment. Our technique comprises the combination of i) semi-automated *in situ* microinjection of chemoattractants or bacteria as local chemotactic stimulus, ii) *in vivo* near-infrared reflected-light oblique transillumination (RLOT) microscopy for the visualization of leukocyte motility and morphology, and iii) *in vivo* fluorescence microscopy for the visualization of different leukocyte subpopulations or fluorescence-labeled bacteria. Leukocyte motility parameters are quantified off-line in digitized video sequences using computer-assisted single cell tracking. Here, we show that perivenular microinjection of chemoattractants [macrophage inflammatory protein-1α (MIP-1α/Ccl3), platelet-activating factor (PAF)] or *E. coli* into the murine cremaster muscle induces target-oriented intravascular adhesion and transmigration as well as polarization and directional interstitial migration of leukocytes towards the locally administered stimuli. Moreover, we describe a crucial role of Rho kinase for the regulation of directional motility and polarization of transmigrated leukocytes *in vivo*. Finally, combining *in vivo* RLOT and fluorescence microscopy in Cx3CR1*^gfp/gfp^* mice (mice exhibiting green fluorescent protein-labeled monocytes), we are able to demonstrate differences in the migratory behavior of monocytes and neutrophils.

Taken together, we propose a novel approach for investigating the mechanisms and spatiotemporal dynamics of subtype-specific motility and polarization of leukocytes during their directional interstitial migration *in vivo*.

## Introduction

The multistep cascade of leukocyte migration into injured or inflamed tissue consists of sequential intercellular adhesion events with endothelial cells [1]. The mechanisms underlying these initial steps of leukocyte rolling and firm adhesion have been studied in detail [1], [2], [3]. In the last decade, new insights have been gained into signaling pathways triggering post-adhesion events such as leukocyte strengthening and intraluminal crawling as well as into the mechanisms mediating leukocyte transendothelial migration (rev. in 2, 3, 4, 5).

After having passed the perivenular basement membrane, transmigrated leukocytes are suggested to locomote within the interstitial tissue in a target-oriented manner [6], [7]. However, the mechanisms underlying leukocyte directional migration within a (patho)physiological microenvironment remain largely unclear. Whilst 2-photon laser scanning microscopy enabled investigations on the dynamics of lymphocyte and dendritic cell migration in the lymph node [8], the majority of previously published studies on interstitial migration of inflammatory cells is based on *in vitro* studies in various 2- and 3-dimensional systems [9]. Thereby, the complex architecture of the interstitial tissue as well as the dramatic phenotypic and functional changes leukocytes undergo during their diapedesis are disregarded [4]. Moreover, directional migration of leukocytes in inflamed “non-lymphatic” tissue is poorly understood. The studying of leukocyte interstitial migration in “non-lymphatic” tissues, however, remains limited because of the induction of diffuse inflammation with “chemotactic chaos” in the interstitial tissue after usage of conventional routes of stimulation such as superfusion or intrascrotal injection of chemoattractants [10], [11], [12]. In addition, adequate *in vivo* models for evaluating leukocyte migration toward bacteria are still lacking. Here, we suggest perivenular microinjection of chemoattractants or bacteria into the murine *M. cremaster* using a microinjection technique in order to induce target-oriented leukocyte migration. Using *in vivo* near-infrared reflected-light oblique transillumination (RLOT) microscopy, we analyzed leukocyte adhesion, transmigration, and interstitial migration upon microinjection with relevant chemoattractants including MIP-1α, PAF, or fluorescent-labeled *E. coli*. We compared the character of leukocyte migration after microinjection of chemoattractants with that induced by a conventional route of stimulation, intrascrotal injection of chemoattractant. Next, we proved our approach by the analysis of the role of Rho kinase for leukocyte motility and polarization *in vivo*. Finally, we combined *in vivo* RLOT and fluorescence microscopy in order to evaluate migration patterns of neutrophils and monocytes in Cx3CR1*^gfp/gfp^* mice.

## Results

### Determination of the optimal distance for microinjection of chemoattractants

**Figure 1:**
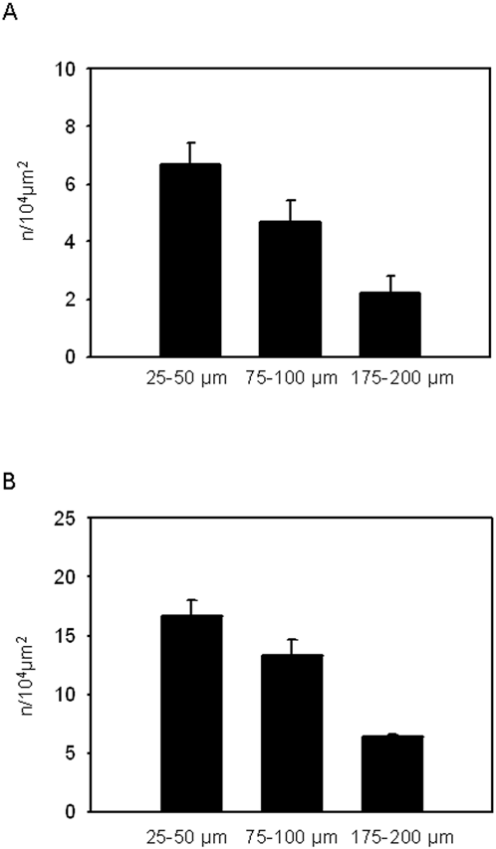
Dependency of leukocyte adhesion and transmigration on the distance of microinjection from the vessel. Leukocyte adhesion (A) and transmigration (B) were analyzed 60 min after microinjection of MIP-1α performed at distances of 25–50, 75–100, and 175–200 µm from the postcapillary venule. Both parameters were observed in ROIs (100×50 µm) along the venule (mean±SEM; n = 3).

In order to establish an optimal protocol for the perivenular microinjection of chemoattractants, we first analyzed the extent of local inflammation in the cremasteric tissue after microinjection of the chemokine MIP-1α performed at three different distances from a venule: 25–50 µm, 75–100 µm, and 175–200 µm. Sixty minutes after microinjection of MIP-1α, leukocyte adhesion and transmigration were analyzed. The highest number of adherent and transmigrated leukocytes was found when the microinjection was performed at a distance of 25–50 µm (Fig. 1). By contrast, the lowest numbers were measured after microinjection performed at a distance of 175–200 µm. Therefore, these data show that for the microinjection of chemoattractants a distance of 25–50 µm from the postcapillary venule is optimal, since the inflammatory response is stronger than after microinjections at the two longer distances analyzed. Consequently, microinjection was performed at a distance of 25–50 µm from the postcapillary venule under investigation in all further experiments.

### Tissue distribution of rhodamine 6G after microinjection

**Figure 2:**
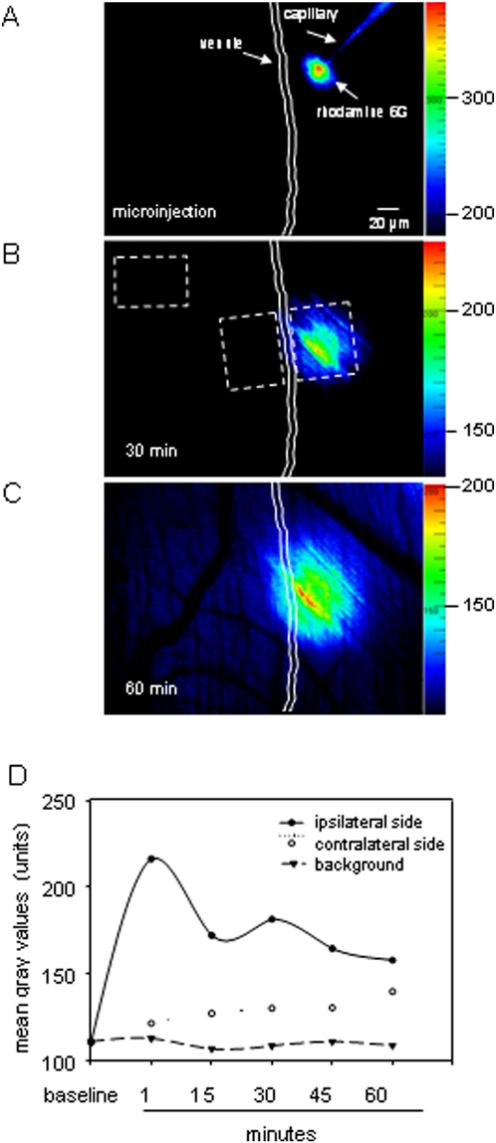
Tissue distribution of rhodamine 6G after microinjection. A-C: *In vivo* microscopy images show tissue distribution of rhodamine 6G at 1 (A), 30 (B), and 60 min after microinjection (C). Three regions of interests 100×75 µm (depicted in B) within the cremasteric interstitial tissue were analyzed: on the vessel side ipsilateral to the microinjection site, on the contralateral side, and in the interstitial tissue at a distance of 350 µm from the site of microinjection (considered as background). Images are shown with different original color scales to emphasize the fluorescent intensity within 60 min after microinjection. The fluorescence intensity was determined within 60 min; the quantitative data are presented in D; n = 7.

In a next step, we sought to evaluate how chemoattractants are distributed within the cremaster tissue after microinjection. In an attempt to answer this question, microinjection (25–50 µm from the venule) of the fluorescent dye rhodamine 6G was performed. Alterations of fluorescence intensity of rhodamine 6G were analyzed within a time period of 60 min in three ROIs (100×75 µm): 1) on the vessel side ipsilateral to the microinjection site, 2) on the contralateral side, and 3) at a distance of 350 µm from the venule (considered as background; Fig. 2B). At baseline conditions prior to microinjection, mean gray values on both the ipsi- and the contralateral side did not differ from background levels (Fig. 2D). Immediately after microinjection, fluorescence intensity was dramatically increased on the vessel side ipsilateral to the microinjection site as compared to baseline levels (Fig. 2A, D). The fluorescence intensity of rhodamine 6G decreased within 60 min after microinjection on the ipsilateral side; however, its levels remained higher in comparison to background values as well as the values measured on the contralateral side (Fig. 2D). Forty minutes after microinjection of rhodamine 6G, the fluorescent dye reached the contralateral vessel side as indicated by a slight elevation of mean gray values (Fig. 2C, D). Hence, these data suggest that microinjection of chemoattractants forms a stable source of chemoattractant in the perivenular region of cremaster muscle with slow distribution in the interstitium during 60 min.

### Leukocyte adhesion and transmigration

**Figure 3:**
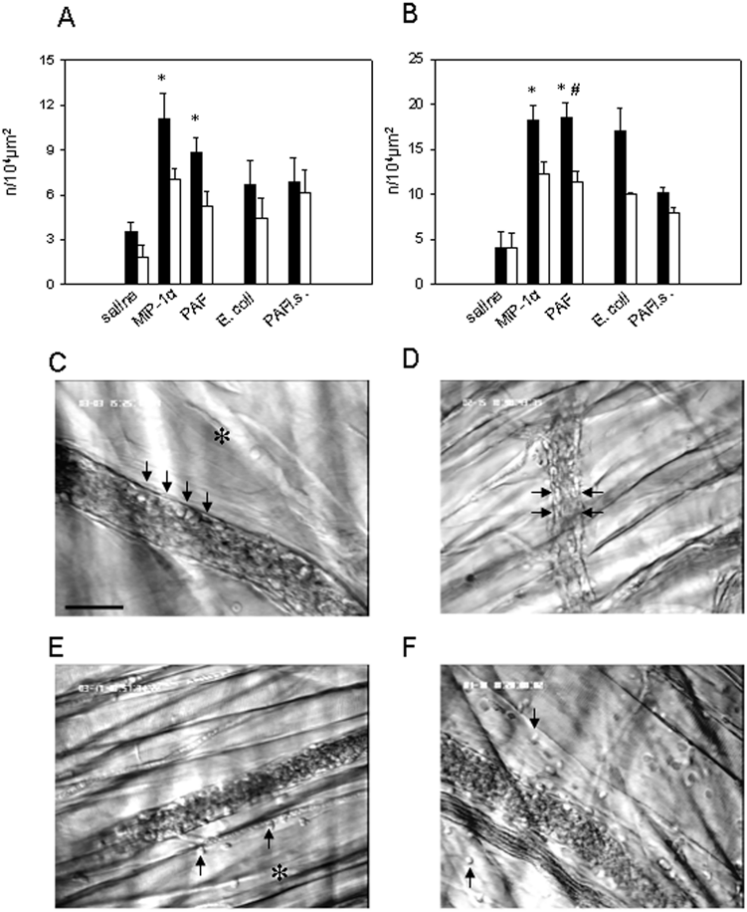
Leukocyte adhesion and transmigration. Numbers of adherent (A) and transmigrated (B) leukocytes at 60 minutes after microinjection of saline, MIP-1α, PAF, or *E. coli*, and after intrascrotal injection of PAF (PAF i.s.) were counted on the vessel side ipsilateral to the microinjection site (black bars) and on the contralateral side (white bars); mean±SEM; n = 6; *<0.05 vs. ipsilateral side, #<0.05 vs. PAF- contralateral side; *E. coli* n = 3; PAF i.s. n = 3. C–F: intravital microscopic images from the murine cremaster muscle demonstrate adherent and emigrated leukocytes (arrows) after microinjection (C and E, respectively) and intrascrotal injection of PAF (D and F, respectively); asterisk shows the site of microinjection; objective magnification 20×; scale bar 25 µm.

In this part of the study, leukocyte adhesion and transmigration were analyzed after microinjection of the chemokine MIP-1α, the phospholipid PAF, and *E. coli* particles. In an additional group, intrascrotal injection of PAF was performed. Leukocyte adhesion and transmigration were observed on the vessel sides ipsi- and contralateral to the microinjection site (Fig. 3). The data show that the numbers of adherent and transmigrated leukocytes were dramatically increased on both vessel sides upon microinjection of MIP-1α, PAF, and *E. coli* particles at 60 min after microinjection as compared to microinjection of saline (Fig. 3). The extent of leukocyte adhesion and transmigration did not significantly differ between the groups undergoing microinjection of MIP-1α, PAF, and *E. coli* (Fig. 3A, B).

Next, we compared leukocyte adhesion and transmigration on the vessel side ipsilateral to the microinjection site with those on the contralateral side (Fig. 3). Upon microinjection of the inflammatory mediators as well as bacteria, 65–70% of all adherent and extravasated leukocytes were localized on the ipsilateral vessel side (Fig. 3A, B, C, E). In contrast, intrascrotal microinjection of PAF was associated with a diffuse character of leukocyte adhesion and transmigration as shown by comparable amounts of adherent and transmigrated leukocytes on both the ipsi- and contralateral vessel sides (Fig. 3A, B, D, F).

### Leukocyte interstitial migration

**Figure 4:**
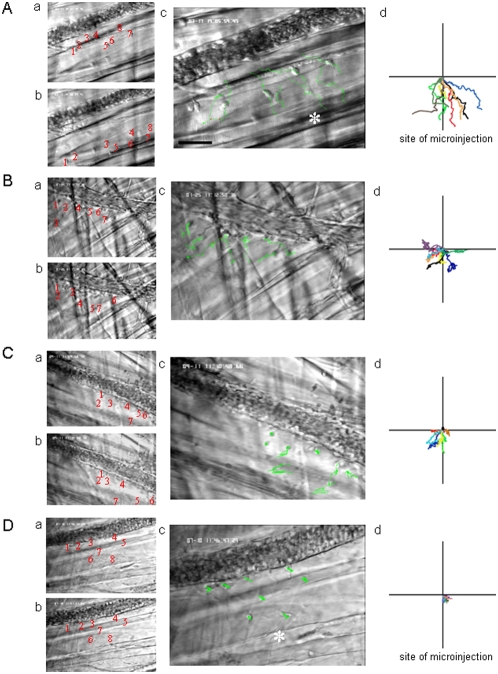
Leukocyte interstitial migration. Interstitially migrating leukocytes were visualized using near-infrared RLOT microscopy (objective magnification 20×) and tracked during 5 min in digitized video recordings using an imaging software. A - leukocyte interstitial migration after microinjection of MIP-1α, B - after intrascrotal injection of PAF, C – upon intrascrotal injection of MIP-1α, and D - after microinjection of MIP-1α followed by superfusion of the Rho kinase inhibitor Y27632. a - red numbers on the intravital microscopic images shows the position of each analyzed leukocyte at the beginning of cell tracking; b - location of the same leukocytes at the end of the cell tracking, respectively; c - green lines on intravital microscopic images and colored lines on the panels (d) show the migration tracks of single leukocytes; asterisks show the site of microinjection, scale bar 20 µm.

**Figure 5:**
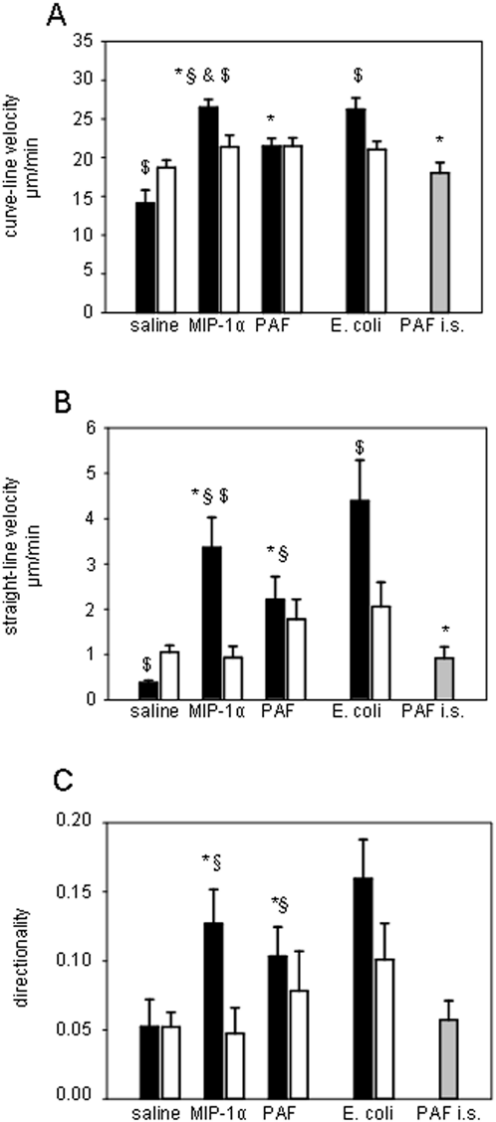
Leukocyte motility. Parameters of leukocyte motility such as curve-line migration velocity (A), straight-line migration velocity (B), and directionality (C) were determined in digitized *in vivo* microscopy video sequences 60 min after microinjection of MIP-1α, PAF or *E. coli* as well as upon intrascrotal application of PAF (PAF i.s.). Interstitially migrating leukocytes (n = 15) were analyzed during 5 min using SimplePCI software. Parameters of leukocyte motility are presented for the vessel side ipsilateral to the microinjection site (black bars), on the contralateral vessel side (white bars), and after intrascrotal application of PAF – (gray bars); mean±SEM; *p<0.05 vs. saline, § p<0.05 vs. PAF i.s., & p<0.05 vs. PAF, $ p<0.05 vs. contralateral side; n = 15; *E. coli* n = 8.

Motility of single interstitially migrating leukocytes was analyzed after microinjection of MIP-1α, PAF, or *E. coli* as well as after intrascrotal injection of PAF. In additional experiments, leukocyte interstitial migration was also studied upon intrascrotal injection of MIP-1α. Microinjection of the inflammatory mediators and bacteria induced interstitial migration of leukocytes (Fig. 4Aa–d; Fig. 5A, B, C). On the ipsilateral side, leukocyte interstitial migration was target-oriented towards the site of microinjection (Fig. 4Aa–d) and characterized by significantly increased curve-/straight-line migration velocity and directionality as compared to leukocytes migrating in saline-treated cremaster muscles or to leukocytes migrating on the contralateral vessel side (Fig. 5A, B, C). Curve-line velocity, a measure of leukocyte migration velocity independent of cell directionality, was just slightly increased upon microinjection of chemoattractants or *E. coli*. By contrast, the effect of microinjection was stronger at the level of straight-line velocity and directionality, parameters that demonstrate how far and how directly leukocytes move toward the stimuli within the time period analyzed. The target-oriented character of interstitial leukocyte migration after microinjection of chemoattractants or bacteria is underlined by the finding that the elevation of straight-line migration velocity and directionality was several times higher than the increase in curve-line velocity (Fig. 5A, B, C). It is worth to be noted that leukocyte motility did not significantly differ between the groups receiving MIP-1α, PAF or *E. coli* in almost all migration parameters, with exception of curve-line migration velocity, which was significantly higher upon microinjection of MIP-1α as compared to that after microinjection of PAF. On the contralateral vessel side, however, the differences in the migration parameters between animals undergoing microinjection and animals from the control group were very weak. Interestingly, in contrast to microinjection, leukocyte interstitial migration was rather random upon intrascrotal injection of PAF (Fig. 4Ba–d) or MIP-1α (Fig. 4Ca–d) as shown by significantly lower straight-line migration velocity and directionality (Fig. 5B, C).

### Effect of the Rho kinase inhibitor Y-27632 on leukocyte motility

**Figure 6:**
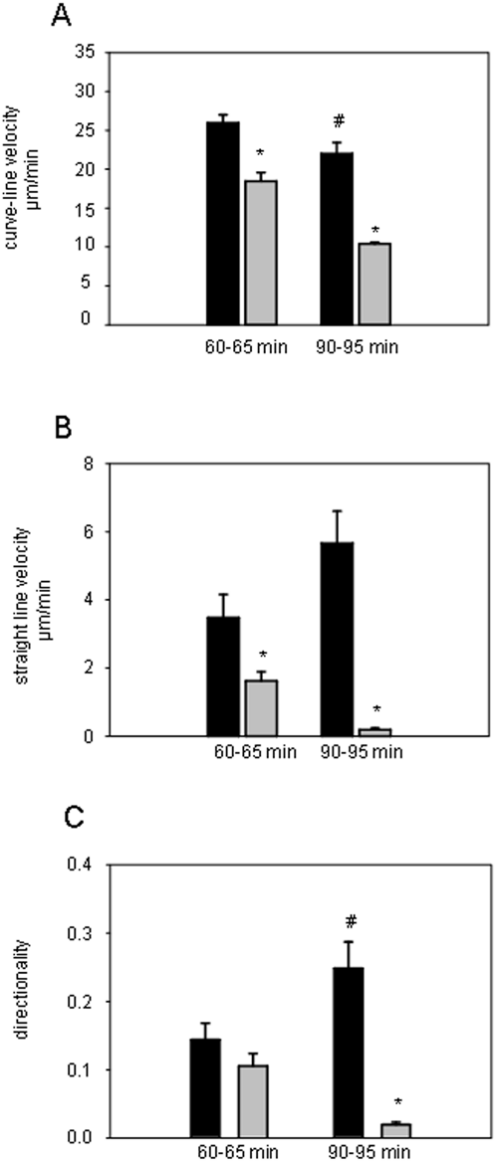
Effect of Rho kinase inhibition on leukocyte motility. Leukocyte curve-line velocity (A), straight-line velocity (B), and directionality (C) were analyzed in the cremaster muscle at 60 min after microinjection of MIP-1α (black bars) followed by superfusion of the Rho kinase inhibitor Y27632 (gray bars) for either 0–5 min or 30–35 min. In control experiments (black bars), the cremaster muscle was superfused with saline upon microinjection of MIP-1α; mean±SEM; *p<0.05 vs. control, # p<0.05 vs. control at 60–65 min; n = 15.

In a separate set of experiments, the effect of the Rho kinase inhibitor Y-27632 on leukocyte motility was analyzed at 60 and 90 min after perivenular microinjection of MIP-1α. After 5 min of superfusion of Y-27632, motility of transmigrated leukocytes was significantly reduced by approximately 45% as compared to controls (Fig. 6) and completely abolished after 30 min of exposure (Fig. 4Da–d; Fig. 6).

### Morphological changes and polarization of interstitially migrating leukocytes

**Figure 7:**
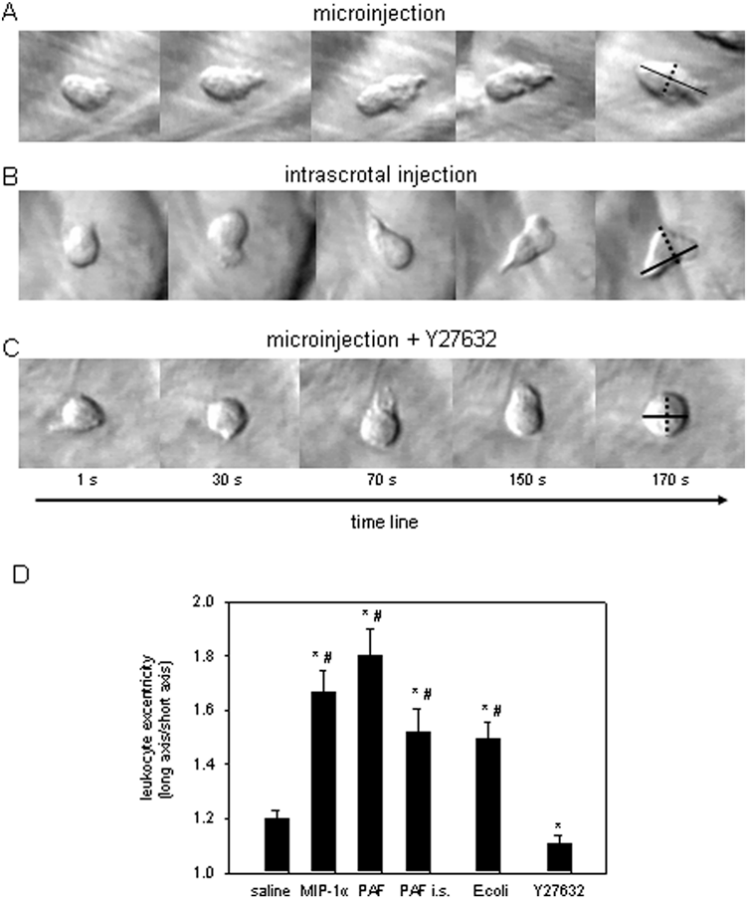
Morphological changes and polarization of interstitially migrating leukocytes. Freeze-frames from *in vivo* microscopy video recordings obtained 60 min after microinjection of PAF (A), 60 min intrascrotal injection of PAF (B), and 60 min microinjection of PAF in combination with superfusion of the Rho kinase inhibitor Y27632 for 30 min (C; objective magnification 40×). A: directional locomotion of a leukocyte towards the attractant with protrusions of lamellipodia at the front and a trailing uropod after microinjection of PAF. B: polarized leukocyte during random migration in the interstitial tissue after intrascrotal injection of PAF. C: blocking effect of Rho kinase inhibitor Y27632 on leukocyte polarization induced by microinjection of PAF. D: leukocyte polarization was measured as cell eccentricity: long axis (shown in black line)/short axis (depicted as interrupted line). Quantitative data of cell eccentricity after microinjection of saline, MIP-1α, PAF, *E. coli*, intrascrotal injection of PAF, as well as after microinjection of PAF with superfusion of Y27632; mean±SEM; *p<0.05 vs. saline, # p<0.05 vs. Y27632; n = 15.

In this part of the study, leukocyte morphological changes and polarization were evaluated after microinjection of chemoattractants or bacteria as well as upon intrascrotal injection of PAF and after Rho kinase inhibition with Y27632. Upon microinjection of MIP-α, PAF, or *E. coli* particles, leukocytes moved toward the applied chemoattractant or bacteria and formed ruffles (Fig. 7). Then, leukocytes adopted an elongated polarized shape change with a contracted tail and lamellipodia protrusions at the front edge (Fig. 7A). Similar shape changes were observed in interstitially migrating leukocytes moving randomly in animals receiving the chemoattractant via intrascrotal injection (Fig. 7B). After application of the Rho kinase inhibitor, however, interstitially migrating leukocytes lost their ability to locomote toward the applied chemoattractant (Fig. 7C). Here, leukocytes became less elongated and more spherical, non-polar with single small protrusions. After microinjection of MIP-α, PAF, or *E. coli*, leukocytes become strongly polarized with an eccentricity of about 1.8 as compared to those after microinjection of saline (Fig. 7A, D). Although the intrascrotal injection of PAF also induced leukocyte polarization, the value of cell eccentricity was significantly less as compared with that after microinjection of PAF (Fig. 7B, D). Application of Y27632 abolished the PAF-induced leukocyte polarization, and the ratio between cell long and short axis was less than 1.2 (Fig. 7C, D).

### Migration patterns of different leukocyte subsets

In the final part of the study, we used a combination of RLOT and *in vivo* fluorescence microscopy in Cx3CR1*^gfp/gfp^* mice in order to analyze the migratory behavior of GFP-positive cells (monocytes) as well as GFP-negative cells (neutrophils) after microinjection of MCP-1.

**Figure 8:**
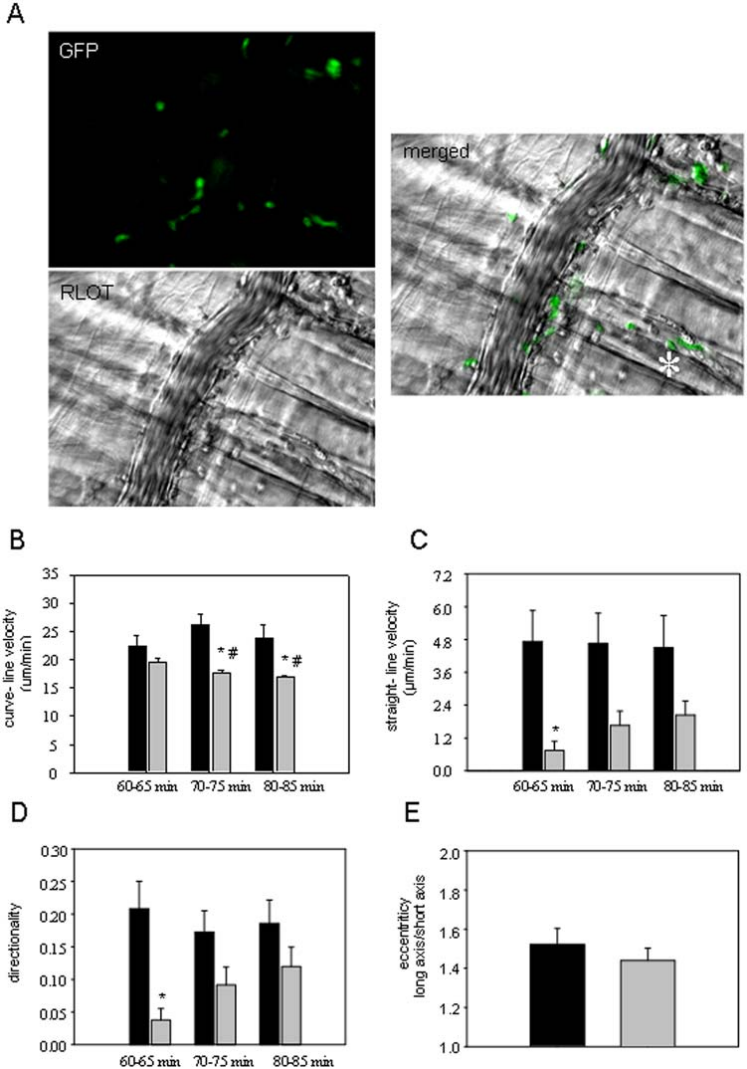
Migration patterns of GFP-positive and GFP-negative cells. A: representative *in vivo* microscopy images show transmigration of GFP-positive (GFP-image) and GFP-negative cells (RLOT-image) at 60 min after microinjection of MCP-1 in Cx3CR1^gfp/gfp^ mice (objective magnification 20×; asterisk shows the site of microinjection). B–E: The motility parameters, curve-line migration velocity (B), straight-line migration velocity (C), and directionality (D) of interstitially migrating, GFP-positive (monocytes, gray bars) and GFP-negative cells (neutrophils, black bars) were analyzed at 60, 70, and 80 min after microinjection of MCP-1 for 5 min, respectively; mean±SEM; *p<0.05 vs. GFP-negative cells; # p<0.05 vs. 60–65 min; n = 5. E: polarization of GFP-negative cells (black bars) and GFP-positive cells (gray bars) analyzed at 70 min after microinjection of MCP-1; mean±SEM, n = 15.

In our experiments, the number of transmigrated monocytes and neutrophils was significantly increased at 60 min after microinjection of MCP-1 (Fig. 8) as compared to baseline conditions (data not shown). Transmigration of leukocytes had a directional character as shown by the distribution of the extravasated monocytes and neutrophils predominantly on the vessel side ipsilateral to microinjection (Fig. 8A). The quantitative analysis of leukocyte interstitial migration demonstrated that perivenular microinjection of MCP-1 induced target-oriented migration of neutrophils within the interstitial tissue as shown by increased curve-line and straight-line migration velocity and directionality (Fig. 8A–D) compared to those after microinjection of saline (Fig. 5A, B, C). Curve-line velocity of neutrophils was significantly reduced at 70 and 80 min as compared to that at 60 min after microinjection of MCP-1. Straight-line migration velocity of neutrophils was comparable between the three analyzed time periods (Fig. 8C). Monocytes, however, did not move toward MCP-1, at least at 60–65 min after microinjection, as indicated by lower levels of curve-line and straight-line migration velocity and directionality (Fig. 8B, C, D). In addition, monocytes displayed a less polarized phenotype at 70 min after microinjection of MCP-1 as compared to neutrophils (Fig 8E), nevertheless these values were not significant. Interestingly, straight-line velocity and directionality of monocytes were slightly increased after 70 min and 80 min after microinjection of MCP-1 as compared to those after 60 min (Fig. 8C, D). These results suggest a delayed chemotactic response of monocytes as compared to neutrophils. Although monocytes displayed a target-oriented character of interstitial migration at 70 and 80 min after microinjection, they migrated slower than neutrophils as presented by lower curve-line, straight-line migration parameters, directionality and polarization (Fig. 8B–E).

### Microhemodynamic parameters and systemic leukocyte counts

To assure intergroup comparability, diameters of analyzed microvessels, centerline blood flow velocity, wall shear rate, and systemic leukocyte counts were measured. No significant differences were detected among the experimental groups (data not shown).

## Discussion

The mechanisms triggering interstitial migration of extravasated leukocytes remain poorly investigated. The vast majority of studies on leukocyte directional migration are performed *in vitro*. However, the mechanisms underlying leukocyte migration differ between *in vitro* and *in vivo* settings [4], [13]. *In vitro* studies do not take into account the phenotypic and functional changes of leukocytes that result from their interactions with endothelial cells and basement membrane during adhesion and transendothelial migration. These changes include increased leukocyte polarization, phagocytosis, release of mediators, enhanced survival as well as upregulation of neutrophil elastase, matrix-metalloproteinases, integrins, etc. [4]. In addition, the pro-emigratory action of chemokines *in vivo* is dramatically different from their capacity to induce chemotaxis *in vitro* [13]. Although 2D substrates were preferentially used for *in vitro* studies on leukocyte chemotaxis, the mechanisms mediating this process seem to be rather different in 2D vs. 3D settings [9], [14]. In contrast to 2D migration, the 3D tissue network confines and mechanically anchors cells from all sides so that they intercalate alongside and perpendicular to tissue structures [14]. In this context, 2D but not 3D leukocyte migration seems to be integrin-dependent [9], [14]. In the present study, we have designed an approach allowing *in vivo* imaging and quantitative analysis of directional subtype-specific leukocyte migration and polarization in inflamed tissue. Our technique comprises the combination of *in vivo* near-infrared RLOT [10] and fluorescence microscopy in the murine cremaster muscle with a microinjection technique for induction of directional leukocyte migration.

Although in chemotaxis assays *in vitro* the mechanisms of leukocyte migration are investigated in the focus of target-oriented cell movement towards a distinct chemoattractant, the study of leukocyte interstitial migration *in vivo* is limited because of induction of diffuse inflammation in the interstitial tissue [10], [11]. An assay allowing the analysis of leukocyte chemotaxis *in vivo* in the murine cremaster muscle has been already described in the literature. It is based on the slow release of chemokines from an agarose gel placed on the interstitial tissue adjacent to a postcapillary venule [15]. A potential disadvantage of this method is that it seems to not consider the distribution of a chemoattractant in the 3D tissue, since the stimulus is applied on the tissue's surface but not in the interstitial milieu. Moreover, although leukocyte chemotaxis towards bacteria *in vitro* and bacterial clearance assays *in vivo* are well described in the literature [16], [6], [17], there is still no *in vivo* approach allowing the analysis of leukocyte directional interstitial migration towards applied bacteria. As a solution, we suggest here to use perivenular microinjection of relevant chemoattractants or bacteria via a microinjection technique. A similar experimental design was previously used in the cremaster model for local administration of chemokines for studying the mechanisms mediating leukocyte rolling and adhesion *in vivo* [18]. Here, we used perivenular microinjection of chemoattractants or bacteria in order to investigate leukocyte interstitial migration. We assume that microinjection of a chemoattractant or bacteria into the interstitial tissue would establish a chemotactic gradient and mimic the local release of mediators during inflammation. Thus, at begin of the study, we evaluated how a microinjected chemoattractant would be distributed in the tissue after microinjection. For this, the distribution of fluorescent dye rhodamine 6G was analyzed after its microinjection. Analysis of fluorescence intensity showed that the concentration of rhodamine 6G was higher on the ipsilateral side up to 60 min after microinjection. However, it is worth to be noted that the distribution of the fluorescent dye may be different from the tissue distribution of a chemoattractant. Furthermore, in additional experiments, microinjection of MIP-1α was performed at different distances from the venule: 25–50 µm, 75–100 µm, and 175–200 µm. We found that a distance of 25–50 µm was optimal for inducing leukocyte migration since microinjections at 75–100 µm and 175–200 µm initiated less leukocyte adhesion and transmigration. Taken together, these observations suggest that microinjection of MIP-1α generates a stable source of chemoattractant with slow diffusion within the interstitial tissue of the cremaster muscle.

In a next set of experiments, we applied different chemoattractants (MIP-1α and PAF) and bacteria (*E. coli*) via microinjection and analyzed leukocyte adhesion, transmigration and interstitial migration. First, we found that microinjection of MIP-1α, PAF, or *E. coli* induced a significant increase in leukocyte adhesion, transmigration, and motility of transmigrated leukocytes as compared to microinjection of saline. Second, we compared the character of leukocyte migration after microinjections of chemoattractants or *E. coli* with that induced by intrascrotal injection. Leukocyte adhesion, transmigration, and interstitial migration were evaluated on the vessel side ipsilateral to the microinjection site and compared with those on the contralateral side. As a result, intrascrotal injection of PAF induced diffuse tissue inflammation without any prevalence of leukocyte adhesion or transmigration on either the ipsi- or the contralateral side. Likewise, leukocyte interstitial migration upon intrascrotal injection of PAF displayed a diffuse character as shown by low levels of leukocyte straight-line velocity and directionality on both vessel sides. In contrast, microinjection of MIP-1α, PAF, or *E. coli* induced leukocyte adhesion and transmigration preferentially on the ipsilateral vessel side. Moreover, upon microinjection of chemoattractants or bacteria, leukocyte straight-line velocity and directionality were several times higher on the vessel side ipsilateral to microinjection as compared to those on the contralateral side. In addition, leukocytes moving directly upon microinjection of chemoattractants/bacteria were more polarized as compared to those after intrascrotal injection of PAF. Taken together, these findings demonstrate that microinjection of inflammatory mediators or bacteria induces directional leukocyte migration toward the chemotactic stimuli. Leukocyte motility was comparable in tissues stimulated with MIP-1α, PAF, or *E. coli*. These data support our previous observation that intrascrotal injection or superfusion of these mediators initiates a comparable extent of leukocyte recruitment and transmigration [19].

How could microinjection of chemoattractants/bacteria induce directional leukocyte migration? On the one hand, chemoattractants administered via microinjection into a perivascular region of the interstitium may diffuse through the extracellular matrix and directly activate the endothelium on the ipsilateral vessel side via G-protein-coupled receptors (GPCRs) on endothelial cells. So called ‘interceptors’ such as DARC (Duffy antigen receptor for chemokines) and D6 have been shown to transport PAF or MIP-1α and present them to the apical side of the endothelium [13], [20]. Bacterial products, mainly N-formylpeptides, can also directly elicit chemotaxis by binding to GPCRs or N-formylpeptide receptor [21]. Alternatively, the endothelium might be stimulated via indirect mechanisms involving the release of mediators derived from cells in the interstitium (e.g., myocytes, fibroblasts, mast cells, smooth muscle cells). Interestingly, glycosaminoglycans have been shown to bind to chemokines and retain them locally in the interstitium, creating a chemotactic gradient and avoiding its rapid distribution in the tissue [22], [23].

Next, we addressed the question of whether specific inhibition of Rho kinase with Y27632 would influence leukocyte interstitial migration in our *in vivo* assay. Rho kinase acts as a key mediator in cytoskeleton reorganization during leukocyte migration and is involved in T cell polarization [24], neutrophil motility [25], as well as chemoattractant-mediated actin assembly during neutrophil chemotaxis [26]. In our study, inhibition of Rho kinase not only attenuated the directional movement and polarization of emigrated leukocytes upon microinjection of chemoattractant but also blocked common migration ability. Therefore, our study provides *in vivo* evidence that Rho kinase plays a critical role for the motility and polarization of emigrated leukocytes toward local chemokine stimulation and supports *in vitro* data from the literature [27], [28]. However, several studies demonstrate that Y27632 induces lamellipodia protrusions in monocytes, inhibits tail retractions and has no effect on forward movement [29], [30]. These controversial data can be explained by stimulus-, tissue-, or leukocyte subtype-specificity of Rho kinase activity.

Finally, we combined *in vivo* RLOT microscopy with fluorescence microscopy in order to analyze MCP-1-induced interstitial migration of monocytes and neutrophils in Cx3CR1^gfp/gfp^ mice in which blood monocytes express GFP [31]. Since neutrophils comprise more than 85% of the leukocyte response to MCP-1 [32], we considered GFP-negative cells as neutrophils. We found that monocytes started their target-oriented interstitial migration later than neutrophils and migrated rather slower than neutrophils. Interestingly, monocytes have been reported to move slower than neutrophils along a stable chemotactic gradient *in vitro* [33], [34]. It seems possible that the ability of neutrophils for fast movement would enable them to accumulate more rapidly at the site of inflammation. Moreover, it has been shown that neutrophils produce chemotactic factors for monocytes under certain inflammatory conditions [35], [36].

In conclusion, we have established an *in vivo* 3D leukocyte chemotaxis assay that opens new avenues for *in vivo* investigations on the mechanisms and spatiotemporal dynamics of target-oriented interstitial migration of single leukocytes.

## Materials and Methods

### Animals

The experiments were performed on male C57BL/6 mice weighing 22 to 28 g (Charles River, Sulzfeld, Germany). For the analysis of migration patterns of monocytes in separate set of experiments, mice expressing green fluorescence protein (GFP) at the locus of the Cx3CR1 gene (Cx3cr1*^gfp/gfp^*) were obtained from European Mouse Mutant Archive (EMMA), Monterotondo, Italy. The animals had free access to tap water and pellet food. All experiments were performed according to German legislation on the protection of animals.

### Reagents and inhibitors

Recombinant murine macrophage inflammatory protein-1α (MIP-1α/Ccl3) and monocyte chemotactic protein-1 (MCP-1/Ccl2) were purchased from R&D Systems® (Wiesbaden-Nordenstadt, Germany). Phospholipid platelet-activating factor (PAF), FITC-labeled *E. coli* particles, and Rho-kinase inhibitor Y27632 were purchased from Sigma Aldrich (Deisenhofen, Germany).

### Surgical preparation

The surgical procedure was made as described in detail elsewhere with slight modifications [37]. Mice were anesthetized by an intraperitoneal injection of ketamine (100 mg/kg) and xylazine (10 mg/kg). The left femoral artery was catheterized in a retrograde manner for the administration of microspheres to the cremasteric vasculature. The right cremaster muscle was exposed through a ventral incision of the scrotum. The muscle was opened ventrally in a relatively avascular zone, using careful electrocautery to stop any bleeding, and spread over the transparent pedestal of a custom-made microscopy stage. Epididymis and testicle were detached from the cremaster muscle and placed into the abdominal cavity. Throughout the surgical preparation and during *in vivo* microscopy, the muscle was superfused with warm buffered saline. Tissue temperature was kept at 37°C using a temperature probe (TFN1093, Ebro, Ingolstadt, Germany) throughout the entire experiment.

### Microinjection of inflammatory mediators and experimental protocol

Leukocyte recruitment and interstitial migration were analyzed in the cremaster muscle after microinjection of 130±30 pl of macrophage inflammatory protein-1α (MIP-1α; 250 nM), the phospholipid mediator platelet-activating factor (PAF; 100 nM), in murine serum opsonized fluorescent-labeled *E. coli* (approxim. 4000), or saline in perivascular regions at a distance of 25–50 µm from a postcapillary venule (6 animals in each group). Single unbranched venules with diameters ranging between 25 and 40 µm and lengths >150 µm were selected for this study. Microinjection was performed under control of the intravital microscope with a water immersion lens (4×/NA 0.12, Leitz, Wetzlar, Germany) using a borosilicate micropipette (tip pressure of 120 hPa (2000 hPa for microinjection of *E. coli*) for 0.5 sec, tip diameter <1 µm) connected to the injecting system involving a semiautomatic micromanipulator (InjectMan NI 2®, Eppendorf, Hamburg, Germany) and a microinjector (FemtoJet®, Eppendorf). A successful microinjection was verified by the observation of visible swelling of the interstitial tissue during injection. The vessel and the surrounding tissue were visualized during a time period of 5 min at baseline conditions before stimulation as well within 60 min after microinjection. In an additional group (n = 3), the character of leukocyte migration was analyzed 60 min after intrascrotal application of PAF (100 nM in 0.3 ml PBS) two hours prior to the *in vivo* microscopic observation. In additional experiments (n = 2), an intrascrotal injection of MIP-1α was performed in order to better compare the character of leukocyte interstitial migration upon intrascrotal injection of MIP-1α with that after microinjection of MIP-1α.

In a separate set of experiments, leukocyte motility was analyzed after inhibition of Rho kinase with a selective inhibitor (Y-27632, 50 µM) [38], [30]. Leukocyte migration was initiated by microinjection of MIP-1α, as described above. Sixty min after microinjection of MIP-1α, the exteriorized cremaster muscle was superfused with Y-27632 for either 5 or 30 min. *In vivo* microscopic analysis was performed upon either 5 min or 30 min of Y-27632 superfusion in two separate groups. For both inhibitor-treated groups, corresponding time controls were performed with saline superfusion.

### *In vivo* microscopy

The set-up for *in vivo* microscopy was centered around an Olympus BX 50 upright microscope (Olympus Microscopy, Hamburg, Germany), equipped for stroboscopic fluorescence epi-illumination microscopy. Light from a 75-W xenon source was narrowed to a near monochromatic beam of a wavelength of 700 nm by a galvanometric scanner (Polychrome II, TILL Photonics, Gräfelfing, Germany) and directed onto the specimen via a fluorescein isothiocyanate (FITC) filter cube equipped with dichroic and emission filters (DCLP 500, LP515, Olympus Microscopy). Microscopic images were obtained with Olympus water immersion lenses [20×/numerical aperture (NA) 0.5 and 40×/NA 0.8] and recorded with an analog black and white charged-coupled device video camera (Cohu 4920, Cohu, San Diego, CA) and an analog video recorder (AG-7350-E, Panasonic, Tokyo, Japan). Oblique illumination was obtained by positioning a mirroring surface (reflector) directly below the specimen and tilting its angle relative to the horizontal plane. The reflector consisted of a round coverglass (thickness 0.19–0.22 mm, diameter 11.8 mm) which was coated with aluminum vapor (Freichel, Kaufbeuren, Germany) and brought into direct contact with the overlying specimen. For measurement of centerline blood flow velocity, green fluorescent microspheres (2 µm diameter, Molecular Probes, Leiden, The Netherlands) were injected via an arterial catheter, and their passage through the vessels of interest was recorded using the FITC filter cube under appropriate stroboscopic illumination (exposure 1 ms, cycle time 10 ms, 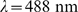), integrating video images for sufficient time (>80 ms) to allow for the recording of several images of the same bead on one frame. Beads that were flowing freely along the vessels were used to determine centerline blood flow velocity (see below).

### Tissue distribution of applied fluorescent dye per time

In an attempt to get information about the character of tissue distribution of injected mediators, microinjection of the fluorescent dye rhodamine 6G (130 pl, 0.05%, Sigma Aldrich) was performed in the cremaster muscle according to the above described technique in a separate set of experiments (n = 7). The distribution of rhodamin 6G in the interstitium was analyzed in the regions of interests using in vivo fluorescence microscopy (excitation: 530 to 560 nm, emission: >580 nm, Olympus). Light from a 75-watt xenon source was narrowed to a near monochromatic beam by a digitally controlled galvanometric scanner (Polychrome II, TILL Photonics, Gräfelfing, Germany). Fluorescence emission was collected by a CCD camera (Sensicam, PCO, Kelheim, Germany) and subjected to digital image analysis (TILL Vision 4.0; TILL Photonics). Spatial dynamics of the fluorescence intensity were measured before and within 60 min after microinjection and expressed as mean gray value [39]. Mean gray values of three regions of interests (ROIs; 100×75 µm) were analyzed: 1) on the vessel side of the postcapillary venule ipsilateral to the microinjection site, 2) on the contralateral side as well as 3) in the interstitial tissue 350 µm from the site of microinjection (considered as background) (depicted in the Fig 2B).

### Microhemodynamic parameters

Centerline blood flow velocity was measured using microspheres administered intraarterially. Quantitative analysis of velocity was performed off-line using CAPImage® by measuring the distance between several images of one fluorescent bead under stroboscopic illumination. From measurement of the vessel diameters and centerline blood flow velocity, the Newtonian wall shear rate [s^−1^] was estimated as 8×[Vb/d], where Vb is the mean blood flow velocity, d is the diameter of the vessel. Mean blood velocity, Vb, was approximated by multiplying the centerline blood velocity by 0.625. The interfacial shear rate is the slope of the velocity profile at the interface of the endothelial surface layer and the vessel lumen, and it was calculated as 4.9×8×[Vb/d], where 4.9 is a mean empirical correction factor [40].

### Parameters of leukocyte recruitment

Quantitative analysis of leukocyte-endothelial cell interactions was performed off-line using CAPImage®. Rolling leukocytes were defined as those moving slower than the associated blood flow and quantified during 30 seconds. Leukocyte rolling flux fraction was determined from video recordings by counting all visible cells passing through a plane perpendicular to the vessel axis and dividing this number by the total leukocyte flux through the vessel, which can be estimated by the product of the systemic leukocyte count, mean blood flow velocity, and estimated vessel cross-sectional area. Firmly adherent cells were determined as those resting in the associated blood flow for more than 30 sec and related to the luminal surface per 100 µm vessel length. Emigrated cells were counted in regions of interests (ROIs) reaching out 75 µm to each side of a vessel over a distance of 100 µm vessel length and are presented per 10^4^ µm^2^ tissue area.

### Single cell tracking of interstitially migrating leukocytes

Intravital microscopic video recordings were transferred into a computer system using a frame grabber. Digital video sequences were analyzed using the imaging software “Simple PCI” (Hamamatsu Corporation/Compix Inc., Cranberry Twp, PA). On each side of analyzed vessel, at least 15 emigrated leukocytes were identified within ROIs and tracked in the perivascular space within a time period of 5 min. Parameters of leukocyte motility such as curve-line and straight-line velocities were automatically calculated by the software. Curve-line/straight-line velocities are the speeds along the curve-line distance or straight-line distance. Curve-line distance is a total (accumulated) distance presented as a line connecting the position of migrating leukocyte at each time point. Straight-line distance represents the shortest line connecting the start and end point of the leukocyte migration track. To quantify the directionality of migration, the chemotactic index (C.I.) was calculated. C.I. is calculated by dividing the distance the cell moved towards the chemoattractant - straight-line distance by the total -curve-line distance the cell moved [41].

### Morphological changes and polarization in interstitially migrating leukocytes

Morphological changes in interstitially migrating leukocytes were evaluated using RLOT microscopy (objective magnification 40×). Randomly chosen single migrating leukocytes were recorded for 5 min after 60 min of chemotactic stimulation performed either by microinjection or intrascrotal injection. Polarization of interstitially migrating leukocytes was analyzed off-line in digitized *in vivo* microscopy images. The major axis and the minor axis of single interstitially migrating leukocytes were measured. Polarization was determined by measuring the eccentricity of the cell which is equal to the ratio of the major axis of the cell (longest straight line that can be drawn across the cell) and minor axis (longest straight line that can be drawn across the cell at 90° to the major axis) [41]. Leukocytes with eccentricity of ≥1.2 were considered as polarized [41].

### Imaging of the migratory behavior of leukocyte subsets

Visualization and quantitative analysis of interstitial migration of monocytes were made in Cx3CR1^gfp/gfp^ mice 60–90 min after microinjection of MCP-1 (115 nM). Cx3CR1*^gfp/gfp^* mice exhibiting green fluorescent protein (GFP)-labeled monocytes were used in this part of the study. The microinjection was performed at a distance of 25–50 µm from the vessel. Interstitial migration of Cx3CR1*^gfp/gfp^*-positive cells and Cx3CR1*^gfp/gfp^*-negative cells was visualized using the combination of *in vivo* RLOT and fluorescence microscopy (objective magnification 20×) and analyzed in perivenular ROI (100×75 µm) on the vessel side ipsilateral to microinjection. Digital *in vivo* microscopy video sequences were analyzed using Simple PCI software. Single cell tracking of extravasated Cx3CR1*^gfp/gfp^*-negative or Cx3CR1*^gfp/gfp^*-positive cells (n = 5 in each group) was performed at 60, 70, and 80 min after microinjection of MCP-1 for 5 min, respectively.

### Statistical analysis

Groups were compared with either ANOVA on ranks followed by Student-Newman-Keuls test (multigroup comparison) or t-test (two-group comparison) using SigmaStat statistic program (Jandel scientific, Erkrath, Germany). Mean values±standard error of the mean (SEM) are given. Differences between experimental groups reaching p value<0.05 were considered as significant.
